# Fabrication of Piezoelectric Electrospun Termite Nest-like 3D Scaffolds for Tissue Engineering

**DOI:** 10.3390/ma14247684

**Published:** 2021-12-13

**Authors:** Thanapon Muenwacha, Oratai Weeranantanapan, Nuannoi Chudapongse, Francisco Javier Diaz Sanchez, Santi Maensiri, Norbert Radacsi, Wiwat Nuansing

**Affiliations:** 1 Institute of Science, School of Physics, Suranaree University of Technology, Nakhon Ratchasima 30000, Thailand; t.muenwacha@gmail.com (T.M.); santimaensiri@g.sut.ac.th (S.M.); 2 Thailand Center of Excellence in Physics (ThEP), Ministry of Higher Education, Science, Research and Innovation, Bangkok 10400, Thailand; 3 Institute of Science, School of Preclinical Sciences, Suranaree University of Technology, Nakhon Ratchasima 30000, Thailand; oratai@sut.ac.th (O.W.); nuannoi@sut.ac.th (N.C.); 4 Center of Excellence on Advanced Functional Materials (CoE-AFM), Suranaree University of Technology, Nakhon Ratchasima 30000, Thailand; 5 Institute for Materials and Processes, School of Engineering, The University of Edinburgh, Robert Stevenson Road, Edinburgh EH9 3FB, UK; s1673949@sms.ed.ac.uk; 6 Research Network NANOTEC—SUT on Advanced Nanomaterials and Characterization, Suranaree University of Technology, Nakhon Ratchasima 30000, Thailand

**Keywords:** 3D scaffold, electrospinning, poly(vinylidene fluoride), tissue engineering, piezoelectric

## Abstract

A high piezoelectric coefficient polymer and biomaterial for bone tissue engineering— poly(vinylidene fluoride-co-hexafluoropropylene) (PVDF-HFP)—has been successfully fabricated into 3D scaffolds using the wet electrospinning method. Three-dimensional (3D) scaffolds have significant advantages for tissue engineering applications. Electrospinning is an advanced method and can fabricate 3D scaffolds. However, it has some limitations and is difficult to fabricate nanofibers into 3D shapes because of the low controllability of porosity and internal pore shape. The PVDF-HFP powders were dissolved in a mixture of acetone and dimethylformamide with a ratio of 1:1 at various concentrations of 10, 13, 15, 17, and 20 wt%. However, only the solutions at 15 and 17 wt% with optimized electrospinning parameters can be fabricated into biomimetic 3D shapes. The produced PVDF-HFP 3D scaffolds are in the cm size range and mimic the structure of the natural nests of termites of the genus *Apicotermes*. In addition, the 3D nanofiber-based structure can also generate more electrical signals than the conventional 2D ones, as the third dimension provides more compression. The cell interaction with the 3D nanofibers scaffold was investigated. The in vitro results demonstrated that the NIH 3T3 cells could attach and migrate in the 3D structures. While conventional electrospinning yields 2D (flat) structures, our bio-inspired electrospun termite nest-like 3D scaffolds are better suited for tissue engineering applications since they can potentially mimic native tissues as they have biomimetic structure, piezoelectric, and biological properties.

## 1. Introduction

In recent decades, there has been extensive research on the fabrication of nanofibers. The simplest and most versatile technique for fabricating continuous nanofibers is the inexpensive electrospinning method [1,2,3,4]. This technique uses a high voltage to fabricate nanofibers from polymer solutions [5].

Fiber scaffolds fabricated with biocompatible polymers with micro- and nanoscale structures have an important role in assisting different aspects of tissue regeneration, such as cell adhesion, proliferation, differentiation, and migration, because these structures can mimic the extracellular matrix (ECM) [6,7,8,9,10,11,12]. Poly(vinylidene fluoride-co-hexafluoropropylene) (PVDF-HFP) is a biocompatible polymer that has appropriate mechanical properties that make it important for several medical applications [13,14,15]. It can be fabricated in special nanofiber structure forms, which are highly porous and have a high surface-to-volume ratio. These exogenous porous characteristics contribute to the potential the material has to mimic the natural ECM, which is required for cell adhesion and cell growth. Another interesting property of the PVDF-HFP polymer is the presence of a piezoelectric response [16,17]. This can enable these nanofiber structures to be used as efficient energy harvesters [18]. Moreover, the PVDF-HFP can be adapted for other applications, such as solar cells [19], lithium-ion batteries [20], and supercapacitors [21]. Mandal et al. have shown the possibility of connecting two PVDF-HFP electrospun nanofiber mats to either double or cancel their output voltage [22]. They have also demonstrated that the output voltage of stacking electrospun nanofibers depends on the polarity of these electrospun nanofibers. With the resulting structures obtained from this research, the 3D nanofiber structures were observed to generate output voltages of far greater magnitudes than those of electrospun as 2D structures (composed of the same nanofibers).

Thus, there is a need for the fabrication of 3D nanofiber structures. There are several techniques that have been used for the creation of 3D electrospun structures, such as gas foaming, self-assembly, hydrogel-integrated fibrous scaffold, and liquid-collecting electrospinning [23,24]. In this work, to achieve this, a liquid-collecting bath electrospinning technique was utilized, as the conventional electrospinning technique has limited capabilities for the fabrication of 3D structures. When electrospun fibers are deposited on a solid collector by stacking layer-by-layer, typically, the fibers cannot grow up from the collector because of the electric force. In the case of liquid-collecting, the electrospun fibers can sink into the liquid and form a 3D structure. However, the fibers should be not dissolved in the liquid-collecting bath [25,26,27]. In addition, the liquid-collecting bath electrospinning is a simple setup, and the liquid collector would suit to fabricate a 3D scaffold for tissue engineering applications. 3D nanofiber structures are similar to the natural ECM structure [28,29,30,31,32,33,34,35]. Therefore, the aim of this work was to fabricate 3D nanofibers structures by using the electrospinning technique with a liquid-collecting bath. This technique uses a liquid reservoir as a collector for the 3D nanofiber scaffold being produced. The fiber size and morphology were investigated by scanning electron microscopy (SEM). The piezoelectricity of the 3D nanofibers was compared with the 2D nanofibers in order to evaluate the energy harvesting potentials. This work also investigated the abilities of the fabricated 3D scaffolds for cell adhesion and migration using NIH 3T3 cells.

## 2. Materials and Methods

### 2.1. Materials

Poly(vinylidene fluoride-co-hexafluoropropylene) (PVDF-HFP) with a molecular weight (MW) of 400,000 g/mol was purchased from Sigma-Aldrich (Singapore). The PVDF-HFP was dissolved in a 1:1 solvent mixture of 99.8% dimethylformamide (DMF) (Sigma-Aldrich, Singapore) and 99.5% acetone (AC) (RCI Labscan, Bangkok, Thailand). For in vitro cell culturing process, NIH 3T3 cells were used. These cells were seeded in a cell growth medium prepared from Dulbecco’s Modified Eagle Medium (DMEM) culture media with 10% fetal bovine serum (FBS) and 1% penicillin/streptomycin (P/S). All culture products were purchased from Gibco (Waltham, MA, USA).

### 2.2. Electrospinning Process

**Figure 1:**
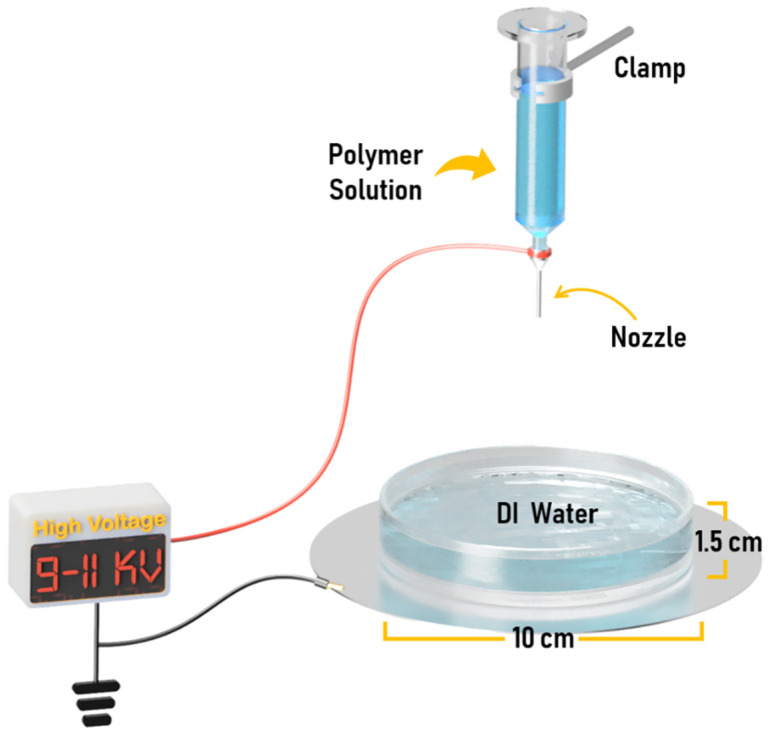
Schematic of the electrospinning method with a water collecting bath.

This work used a liquid bath as a collector for electrospinning to fabricate 3D structure nanofiber structures (see Figure 1 and Figure S1, Supplementary Materials). Using a liquid collector is an easy method for fabricating 3D nanofiber structures by electrospinning [36]. Deionized water (DI water) was used as the liquid in the collecting bath. The polymer solution was prepared by dissolving the polymer in a 1:1 ratio of DMF and AC, adjusting the polymer concentration to 10–20 wt%. This solvent system had the correct vapor pressure and viscosity for the electrospinning process to be running without a syringe pump, relying only on gravity. The electrospinning setup consisted of a 10 mL syringe with the plunger removed from it and an 18G blunt needle, which was placed on a clamp vertically. The liquid collecting bath was set up by placing a glass Petri dish with a diameter of 10 cm and a height of 1.5 cm on a copper plate. A distance between the tip of the nozzle and the water surface was fixed at an optimized distance of 5 cm (see a result when the distance was changed to 10 cm in Figure S2, Supplementary Materials). The schematic of the electrospinning setup is shown in Figure 1. In this work, the solution ejection from the tip of the nozzle was performed without a syringe pump and relying only on gravity. Under this condition, the polymer solution was filled in a 3 mL syringe, but it was filled up to 1 mL per experiment. This method allowed the flow rate to be kept constant by gravity. The applied high voltage was adjusted between 9–11 kV.

### 2.3. Scanning Electron Microscopy

For the fiber and cells morphology analysis, the electrospun nanofibers were coated with 15 nm of gold with a gold sputtering machine (JFC-1100E, JEOL, Tokyo, Japan), and then the nanofiber morphology and the cell attachment were observed by a scanning electron microscope (SEM, JSM- 6010LV, JEOL, Tokyo, Japan) with 10 kV acceleration voltage. Diameters of the electrospun nanofibers were measured manually by more than 150 fibers per sample by importing SEM images into ImageJ analysis software (National Institutes of Health, MD, USA).

### 2.4. Generator Assembly for Piezoelectric Response Testing of the 3D Nanofiber Scaffolds

**Figure 2:**
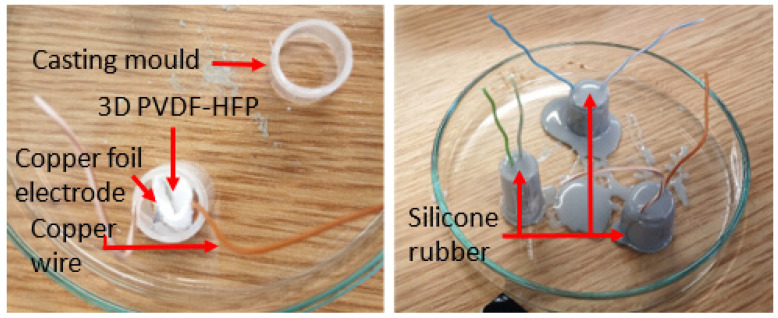
Generator assembly using the PVDF-HFP 3D fibers structure as the active core material. The image on the left shows the mold used for casting the generators and how the generator core, already fitted with electrodes and copper wire, is placed on the mold before casting the silicone rubber. In the image to the right, generators are shown during the curing process after having poured the silicone rubber inside the molds.

When subjected to a mechanical stimulus, some PVDF structures will display an electrical response with an amplitude dependent on several properties such as the predominant crystal phase and the object geometry. To evaluate and compare the piezoelectric response of the PVDF-HFP 3D structures obtained in this study, a 1 cm^3^ fragment of the 3D nanofiber scaffold and a 12 cm^2^ cut of a flat PVDF-HFP fiber mat were used as the active cores of a piezoelectric generator. Thin, sticky copper foil and copper wires were attached to the cores to serve as electrodes (see open voltage response as a function of the electrode properties in Figure S4, Supplementary Materials). Then, the structures were coated in a flexible, condensation cure silicone rubber (CS25, Easy Composites Ltd., Stoke-on-Trent, UK), which would serve the purpose of protecting the cores and providing an electromechanical interface for the generators. Encapsulating the active core material and electrodes in silicone rubber also ensures that the electrical response observed can be mainly attributed to the piezoelectric effect. The silicone rubber keeps the core and electrodes in place, minimizing the incidence of triboelectric charging, which mainly occurs by friction or charging effects from the temporary separation of the materials used to build the generator. Figure 2 shows how the 3D fiber structures were used to assemble the generators.

After assembly, the generators were connected to a 3 MΩ load and subjected to mechanical stimulation in the form of an impacting 100 g weight dropped from a height of 5 cm into the surface of the generators. The piezoelectric response of the generators was evaluated by recording the voltage observed across the terminals of the resistive load and the current generated on it. Measurement of the output voltage signal was done by setting up an operational amplifier in buffer configuration. This would allow for measuring the voltage signal without causing any loading effects. The testing circuit also had a transimpedance amplifier (TIA) which acted as a virtual ground for the system, allowing for the conversion of the current sourced by the generator into a voltage signal without the need of using a sense resistor in series with resistive load. These arrangements allowed the voltage and current responses to be recorded simultaneously. The same experimental procedure was repeated to evaluate the generator response under different resistive load conditions. Load resistors ranging from 1.5 MΩ to 9 MΩ, in 1.5 MΩ increments, were used for this experiment. An RTB 2004 digital oscilloscope (Rohde and Schwarz, Munich, Germany) was used for capturing the output signals.

After connecting the probes, generators, and resistive loads, the testing weight was dropped on the generator surfaces. The weight should ideally not be allowed to impact the surface of the generator more than once in order to record the response of the samples to an initial impulse only. The trials were repeated up to five times for each of the assembled generators. The signals were processed and conditioned using spreadsheet software and MATLAB (Mathworks, Natick, MA, USA).

### 2.5. Surface Modification

In order to enable cell adhesion to the nanofiber scaffold, it needs to be hydrophilic [37] before the cell culture process can take place. As the 3D nanofiber scaffold produced from PVDF-HFP is superhydrophobic, it is only suitable for cell culturing after modification of the surface. This work used oxygen plasma treatment to modify the surface properties of the nanofiber scaffolds. The applied plasma power was 300 W for a treatment time of 120 s and an oxygen flow rate of 120 mL·min^−1^; this parameter has been optimized in another research [38].

### 2.6. In Vitro Cell Culture

The fabricated, surface-modified 3D scaffolds were sterilized under ultraviolet light for 30 min. Thereafter they were soaked in 70% ethanol for 30 min and then washed with phosphate-buffered saline (PBS) solution for 5 min, which was repeated three times. The final step consisted of having the scaffolds soaked in a cell culture medium overnight to facilitate cell attachment onto the 3D scaffolds. For the cell culture process, the pre-wetted 3D electrospun scaffolds were placed in 12-well plates. Then, the NIH 3T3 cells were seeded on the scaffold at a density of 2 × 105 cells per well and incubated at 37 °C with 5% humidified CO_2_ for 24 h.

After three days of cell culture, the 3D electrospun scaffolds were washed twice with PBS for 10 min, and then the cells on the scaffolds were fixed with 2.5% glutaraldehyde (0.5 mL/well) for 1 h at room temperature. After that, the 3D electrospun scaffolds were gently washed twice with PBS for 10 min, and the cells were dehydrated through a series of alcohol exposure. Finally, the critical point drying, which is an established method of dehydrating biological tissues prior to SEM examination [39], was used to maintain the cell morphology.

## 3. Results and Discussion

### 3.1. Fabrication of 3D Nanofiber Scaffolds

**Figure 3:**
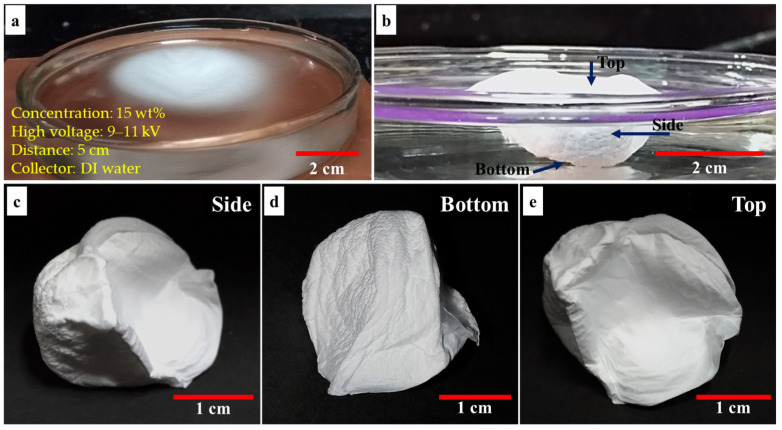
The PVDF-HFP fibers: (**a**) floating on the water and slightly collapsed during the electrospinning process, (**b**) was pulled into the water by weight of stacking fibers, (**c**–**e**) each side of 3D nanofibers scaffold after electrospinning.

During the electrospinning procedure, when the PVDF-HFP nanofibers reach the water surface, they will not dip into the water due to being a superhydrophobic polymer. Instead, the surface tension of the water will lead to the fibers collected as a 2D sheet floating on the water surface at the beginning of the electrospinning process (Figure 3a). As the fibers stack layer-by-layer on the water surface, the weight of fibers on the water surface will steadily increase, and at one point, the weight of the nanofibrous structure will overcome the surface tension of water, causing the structure to be gradually pushed into the water (Figure 3b). This method enables the formation of hydrophobic polymers as 3D nanofibrous structures (Figure 3c–e).

**Figure 4:**
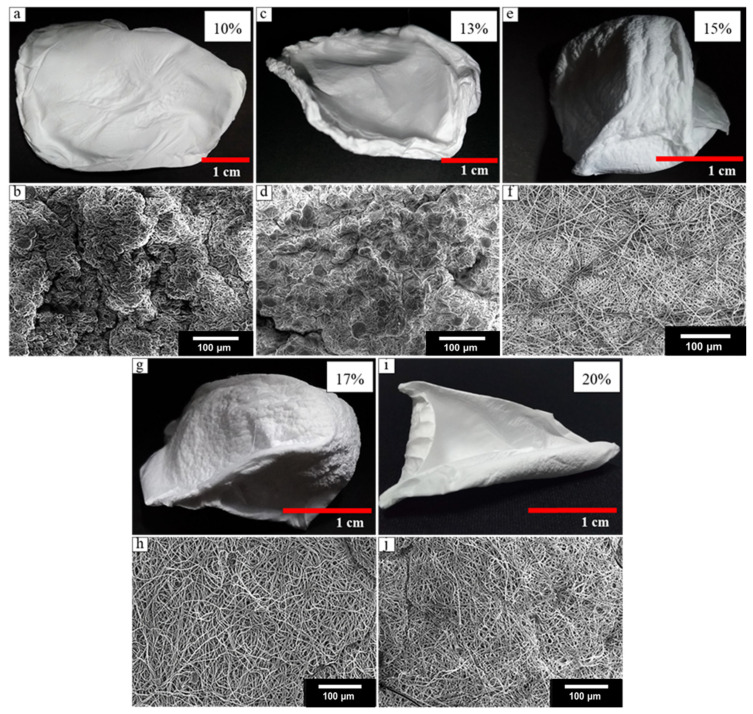
Optical and SEM images show electrospinning results of PVDF-HFP at various concentrations: (**a**,**b**) 10 wt%, (**c**,**d**) 13 wt%, (**e**,**f**) 15 wt%, (**g**,**h**) 17 wt%, and (**i**,**j**) 20 wt%.

To investigate the concentration effects of the PVDF-HFP solution on the construction of the 3D structure nanofibers, the solution concentrations were varied between 10–20 wt% (10, 13, 15, 17, and 20 wt% concentrations were investigated). As shown in Figure 4a, the 10 wt% solution did not produce a 3D scaffold structure. The deposited fibers spread widely on the water surface and did not sink into the water surface. The average diameter of the obtained fibers was 441 ± 18 nm, and the fiber showed the formation of beads and agglomerated fibers (Figure 4b). The solution viscosity has a great effect on bead formation. The polymer solution jet gradually shrinks into a sphere shape to achieve the smallest surface area due to surface tension. If the polymer solution has low viscosity (low concentration), this effect is easy to occur. It is possible that the amount of the beads inside the fibers made the sample float on the water surface as too much air was trapped in this nanostructure. Similarly, when the solution concentration was increased to 13 wt% of PVDF-HFP, the beads were still present, and the electrospun sample was not capable of producing the 3D fiber structure. The fibers that were produced from 13 wt% PVDF-HFP solution concentration had a lower number of beads within the fiber than the fibers that were produced from the 10 wt% solutions (shown in Figure 4b,d). However, they had an average diameter 451 ± 16 nm, which was similar to the average fiber diameter obtained from the 10 wt% solution concentration (Figure 4c,d). It is thus assumed that the presence of beads in the sample prevents the formation of 3D structures with the solution bath method.

The 3D structure nanofibers can be successfully produced with a 15 wt% solution (Figure 4e,f). The next step was to evaluate if higher concentrations would also yield a final product in the form of a 3D structure. Using a solution with a polymer concentration of 17 wt% (Figure 4g,h) also resulted in a 3D structure similar to those seen for the 15 wt% solutions. Thus, the external structure was not different.

As shown in Figure 4i,j, the use of a 20 wt% solution did not successfully produce a 3D nanofiber structure. These fibers have a larger area than that of the product obtained from using the 15 and 17 wt% solutions. During the electrospinning process, the fibers did not stack into layers, ultimately collapsing into the water. The average diameter of these fibers was 1277 ± 32 nm, which was not significantly different from that seen from using the 17 wt% solutions. Although those fibers had similar average diameters, the 3D structure was not produced when using a solution with a polymer concentration of 20 wt%. A possible explanation is that the solution with 20 wt% concentration had a higher viscosity than of 17 wt% concentration during the electrospinning process, resulting in a lower flow rate and a slower layer deposition than those observed for the 15 and 17 wt% solutions. Given those observations, the electrospinning setup for this condition was adjusted to use a lower applied voltage than that chosen for the 15 and 17 wt% solution electrospinning. With the lower flow rate and lower applied voltage (low electric force), the whipping jet that forms during the electrospinning process has a low area of spinning, and the fibers that stack like a sheet on the water surface slowly collapse into the water. Therefore, the 3D build-up process is not possible for the 20 wt% solution samples under those set conditions.

In conclusion, for the solution bath electrospinning method, the PVDF-HFP solution concentrations need to be in the range of ~15–17 wt% where the nanofibers show no beads in the fiber structures to enable the formation of the 3D structures (see Table S1, Supplementary Materials). Thus, the solution concentration is a key parameter for the production of 3D nanofiber structures.

**Figure 5:**
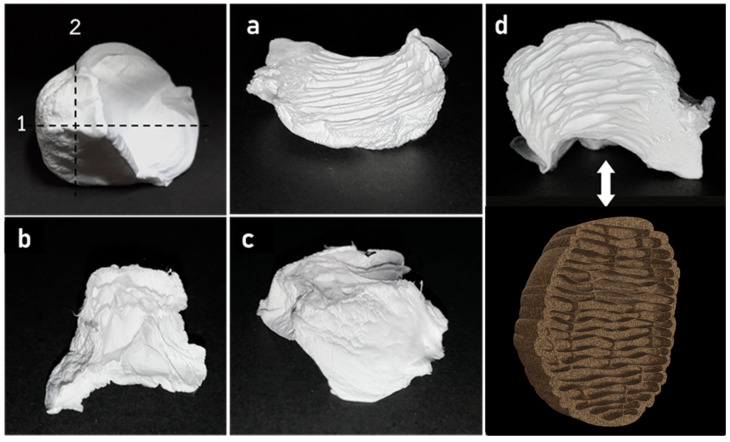
Cross-section of 15 wt% PVDF-HFP 3D nanofiber scaffold: (**a**) line number 1, (**b**,**c**) line number 2, and (**d**) compared with a 3D model of termite nest.

The 3D structures maintain their original shapes over time, are flexible, and can recover their original shape after being pressed or squeezed by hand. The height and width of this fiber structure are around 1.5 cm × 1.5 cm. The 3D fiber structures have many pores inside. Figure 5 shows the cross-section images from the inside of this fiber structure. The structure of the resulting product resembles natural termite nests (Figure 5d and see natural termite nests in [40]). This structure can repeat with this process, see in Figure S3, Supplementary Materials.

**Figure 6:**
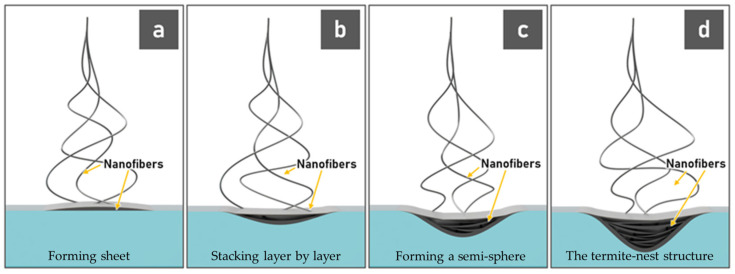
Schematic drawing of the termite nest structure formation process: (**a**) forming sheet, (**b**) stacking layer by layer, (**c**) forming a semi-sphere, and (**d**) the termite-nest structure.

The construction of these 3D fiber structures was observed to consist of the fibers reaching the water surface and forming sheets that stack layer by layer until the structure grows and overcomes the surface tension of water. Then the stacked structure started sinking into the water, resulting in curves of fiber layers, forming a semi-sphere. The fibers that were ejected later were stuck to the structure at the edges of the previous fiber sheet. Therefore, there was a regular spacing in the 3D fiber scaffold, leading to the termite-nest structure. A schematic drawing of the termite nest structure formation process is shown in Figure 6.

**Figure 7:**
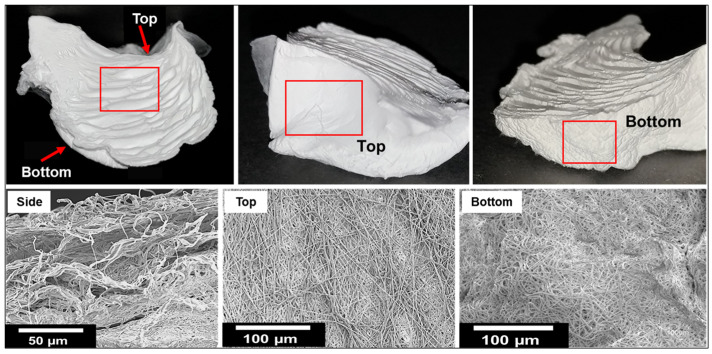
SEM images of the 3D structured nanofiber-based samples.

The location of layers affected the characteristics of the fibers present in each layer. As shown in Figure 7, the morphology of the fibers that were fabricated with the 15 wt% solutions was different for each region of 3D fibers (top and bottom). The fiber layers at the bottom of the structure, which were most in contact with the water surface, were not similar to the fiber layers at the top of the structure, which had no contact with water. The alignments of fibers of the two regions were also different. The average nanofiber diameter was 802 ± 23 nm.

**Figure 8:**
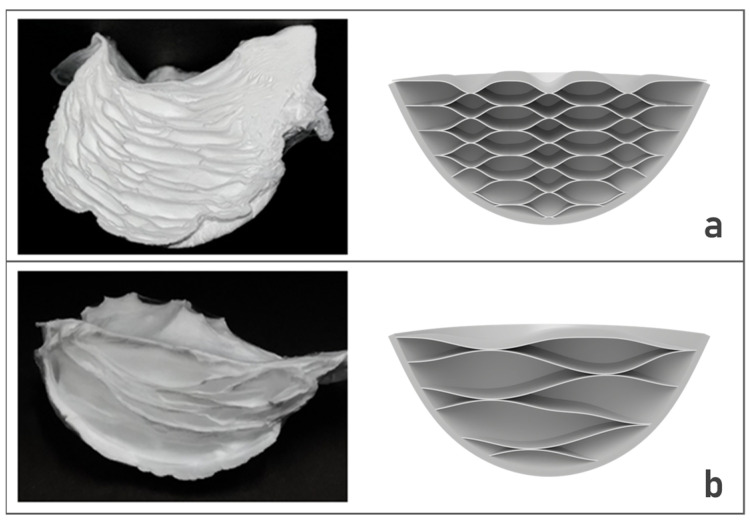
Comparison of (**a**) 15 wt%, and (**b**) 17 wt% 3D samples inside structure and illustration of their cross-section structure by drawings. The 17 wt% 3D structure has larger and longer (in the horizontal axis) pores inside the structure than the 15 wt% 3D sample.

The cross-section of these fibers was determined by the polymer concentration. The cross-section of 3D nanofibers that were produced with the 17 wt% solutions showed larger pores inside the structure and greater length than those observed for the 15 wt% 3D fiber structures (Figure 8). The number of pores per area of 15 wt% fiber is 15 pores/cm^2^, and 17 wt% fiber is 4 pores/cm^2^. The average fiber diameter with the 17 wt% solution concentration was 1256 ± 43 nm, which is significantly larger than that of the 15 wt% 3D fibers structures (802 ± 23 nm). This indicates that the fiber diameters were increased with the increase of the solution concentration. In addition, the fibers with larger diameters have more contact surfaces than the smaller fibers. Therefore, during the electrospinning process, the 17 wt% fibers were located on the water surface and spread with a larger area than 15 wt%, leading to the slower sink into the water. Consequently, the pores inside 3D nanofiber are larger when concentration is increased.

### 3.2. Piezoelectricity of 3D Nanofibers

**Figure 9:**
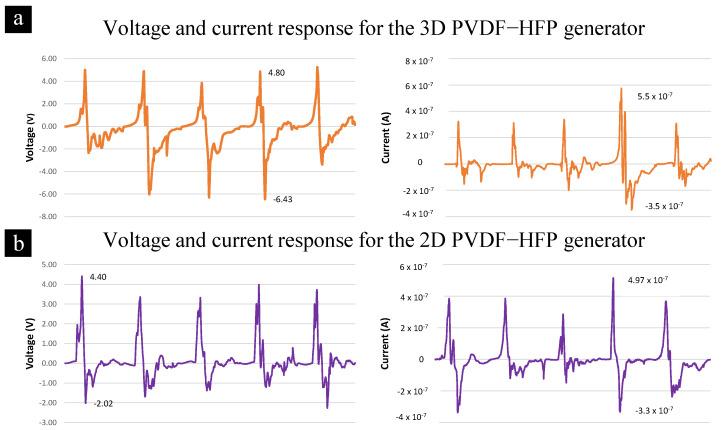
The output voltages and current response for 3 MΩ resistive load of (**a**) 2D and (**b**) PVDF−HFP 3D nanofibers.

The electric response of the 3D and 2D PVDF-HFP nanofibers to a mechanical impact is shown in Figure 9. The response observed for each mechanical impact shows the peaks attributed to the compression and relaxation of the generator, followed by additional residual peaks that occur as the generator is allowed to vibrate freely after the impact event. The 2D nanofibers can generate output max/min voltages of 4.40 V/−2.02 V and max/min current of 0.49 µA/−0.33 µA. The 3D nanofibers can generate output max/min voltage of 4.79 V/−6.43 V and max/min current of 0.55 µA/−0.35 µA. This result shows that the 3D nanofiber generator cores can generate maximum output voltages and currents that are significantly higher than those obtained from using a 2D nanofiber core of the same material. Thus, these 3D nanofibers have potential uses for energy generation applications.

To further explore the response of the 3D PVDF-HFP generator, different resistive loads were tested under similar experimental conditions. The peak-to-peak voltages and currents were obtained from identifying the maximum and minimum values for each recorded signal. Increasing the load resistance resulted in a consistent reduction of the *I_PP_* values, as would be expected. However, while the output *V_PP_* of the generator was expected to increase, this was not the case for some of the recordings. This could occur because of the 100 g weight not striking in exactly the same place when it was dropped on the generator for each trial.

One method to evaluate the output power of the generators is to obtain the root mean square (RMS) of the recorded voltages and currents. The *V*_*RMS*_ and *I*_*RMS*_ values describe an alternating current signal in the context of direct current systems, being equivalent to the constant voltage and current values observed on an alkaline battery, for example. An estimate of the average power (*P_AVG_*) can be obtained by multiplying *V_RMS_* and *I_RMS_*_._ The highest *P*_*AVG*_ was observed when the resistive load was 3 MΩ, indicating that maximum power extraction can be achieved when the generator is connected to resistive loads in the vicinity of this value. Contrary to what was observed for the peak-to-peak values, there was a clear and consistent increase in *V_RMS_* and decrease in *I_RMS_* as the resistive load increased, with only one exception for the *V_RMS_* value obtained when the resistive load was 3 MΩ. However, even if the *P_AVG_* value estimated for this resistive load was considered an outlier, the maximum *P_AVG_* would still occur for an unknown resistive load between 1.5 MΩ and 4.5 MΩ if the corresponding *P_AVG_* values are taken into consideration. The results of this experiment are summarized in Table 1.

### 3.3. Cell Morphology and Attachment

**Figure 10:**
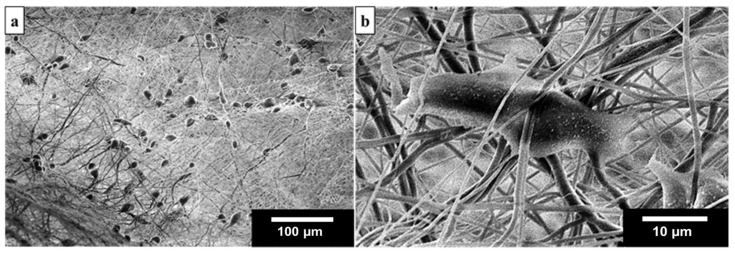
(**a**) SEM image showing the NIH 3T3 cells attached to the 15 wt% 3D nanofiber scaffold, and (**b**) SEM image of an NIH 3T3 cell inside the 3D nanofiber structure.

The 3D nanofiber scaffolds that were modified by using oxygen plasma were found to be adequate to be used for cell culturing (see Figure S4, Supplementary Materials). As shown in Figure 10, these SEM images present the morphology of NIH 3T3 cells after 1-day in vitro culture. The cells spread and attached to the fibers inside the 3D nanofiber-based scaffolds (can grow at the bottom of the pore in fibers structure), cells were grown on the surface of fibers with an average cell density of around 460 cells per mm^2^. Thus, the resulting 3D nanofiber scaffold, which has a structure that resembles a termite nest, can support cell adhesion and has promising potential for medical applications.

## 4. Conclusions

3D nanostructured materials that mimic natural termite nests have been presented in this study. The liquid collector bath electrospinning technique was used with DI water as liquid to successfully fabricate these 3D structures of biocompatible PVDF-HFP nanofibers. The produced 3D nanofibers showed a piezoelectric response and can generate output voltages and currents of greater magnitude than an arrangement of 2D nanofiber mats of the same polymer. This offers open-ended possibilities for scaffold fabrication for energy harvesting applications. Cell culture studies using the NIH 3T3 cell line showed that the cells were attached to the 3D nanofiber scaffolds after a 1-day in vitro culture. The scaffolds produced in this study are potentially suitable for cell adhesion, thus are promising for biomedical applications.

## Figures and Tables

**Table 1:** Electrical output characteristics of the 3D PVDF-HFP generator when a 100 g weight is dropped on its surface from a height of 5 cm, under different resistive load conditions.

Resistive Load (MΩ)	1.5	3.0	4.5	6.0	7.5	9.0
*V_PP_* (V)	2.86	4.79	4.32	4.22	4.36	5.15
*V_RMS_* (mV)	288	533	479	529	520	605
*I_PP_* (µA)	1.85	1.54	0.93	0.67	0.56	0.55
*I_RMS_* (nA)	191.1	172.6	105.3	85.6	68.0	65.8
*P_AVG_* (nW)	55.0	92.0	50.4	45.3	35.3	39.8

## Data Availability

The data presented in this study are available upon request from the corresponding author.

## Supplementary Materials

The following are available online at https://www.mdpi.com/article/10.3390/ma14247684/s1, Figure S1: Illustration of the 3D electrospinning setup with a water-collecting bath, Table S1: The production of PVDF-HFP 3D nanofibers structure, Figure S2: The fibers covering the water surface when the distance between the tip of the needle and the water surface was changed from 5 to 10 cm, Figure S3: The termite nest-like 3D scaffold can be reproduced, Figure S4: The water drops on the PVDF-HFP 2D and 3D nanofiber, Figure S5: Open voltage response as a function of electrode attachment to the piezoelectric fibers.
