# Supplementary material for: Fabrication of Piezoelectric Electrospun Termite Nest-like 3D Scaffolds for Tissue Engineering
Source: Materials (Basel). 2021 Dec 13;14(24):7684. doi: 10.3390/ma14247684 (PMC8708465; doi:10.3390/ma14247684)
Supplement: Supplementary file 1 [file materials-14-07684-s001.zip › materials-1469983-supplementary.pdf]

materials

Supplementary Materials

Fabrication of Piezoelectric Electrospun Termite Nest-like 3D Scaffolds for Tissue Engineering

Thanapon Muenwacha [1,2], Oratai Weeranantanapan [3,4], Nuannoi Chudapongse [3,4], Francisco Javier Diaz Sanchez [5], Santi Maensiri [1,4,6], Norbert Radacsi [5,*] and Wiwat Nuansing [1,4,6,*]

[1] Institute of Science, School of Physics, Suranaree University of Technology, Nakhon Ratchasima 30000, Thailand; t.muenwacha@gmail.com (T.M.); santimaensiri@g.sut.ac.th (S.M.)
[2] Thailand Center of Excellence in Physics (ThEP), Ministry of Higher Education, Science, Research and Innovation, Bangkok 10400, Thailand
[3] Institute of Science, School of Preclinical Sciences, Suranaree University of Technology, Nakhon Ratchasima 30000, Thailand; oratai@sut.ac.th (O.W.); nuannoi@sut.ac.th (N.C.)
[4] Center of Excellence on Advanced Functional Materials (CoE-AFM), Suranaree University of Technology, Nakhon Ratchasima 30000, Thailand
[5] Institute for Materials and Processes, School of Engineering, The University of Edinburgh, Robert Stevenson Road, Edinburgh EH9 3FB, UK; s1673949@sms.ed.ac.uk
[6] Research Network NANOTEC—SUT on Advanced Nanomaterials and Characterization, Suranaree University of Technology, Nakhon Ratchasima 30000, Thailand
* Correspondence: N.Radacsi@ed.ac.uk (N.R.); w.nuansing@g.sut.ac.th (W.N.);
Tel.: +44-(0)-131-651-7112 (N.R.); +66-(0)-6254-55630 (W.N.)

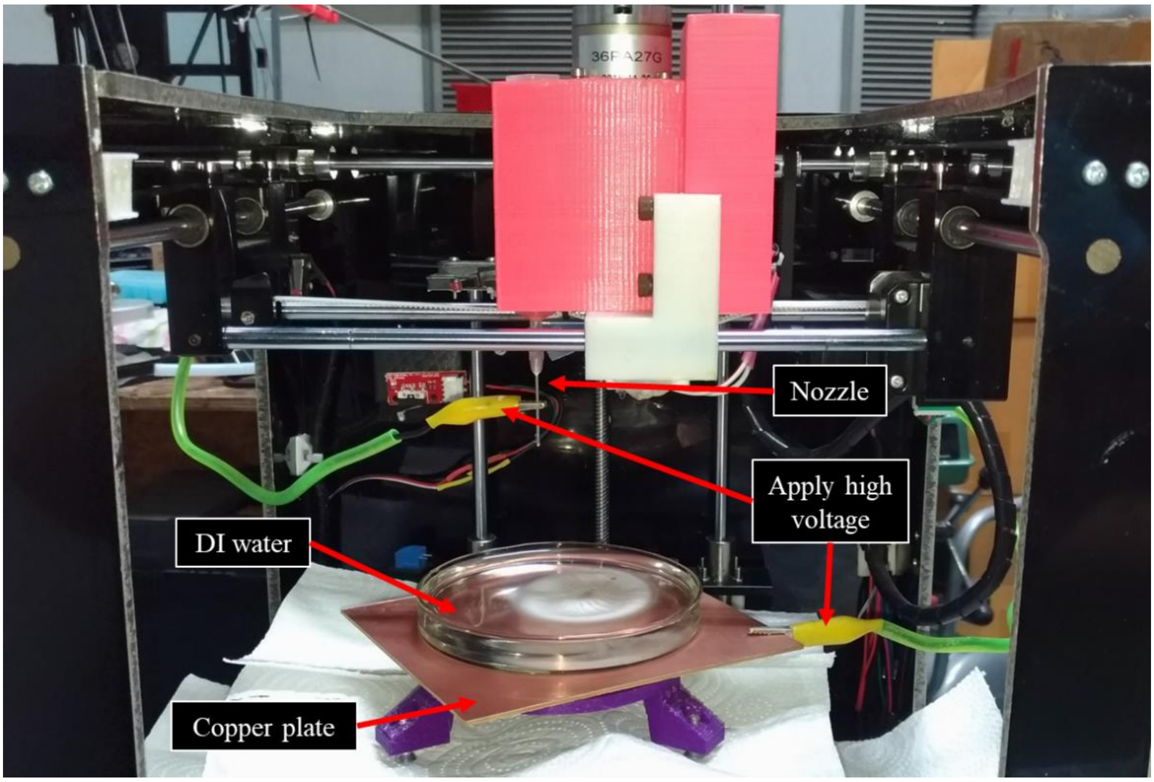

Figure S1. Photograph of the 3D electrospinning setup with a water-collecting bath.

Table S1. The production of PVDF-HFP 3D nanofibers structure.

Concentration (wt%)	HV (kV)	Diameter (nm)	Beads	3D structure
10	15.5	441 ± 18	Yes	No
13	12.8	451 ± 16	Yes	No
15	10	802 ± 23	No	Yes
17	9.2	1256 ± 43	No	Yes
20	7.1	1277 ± 32	No	No

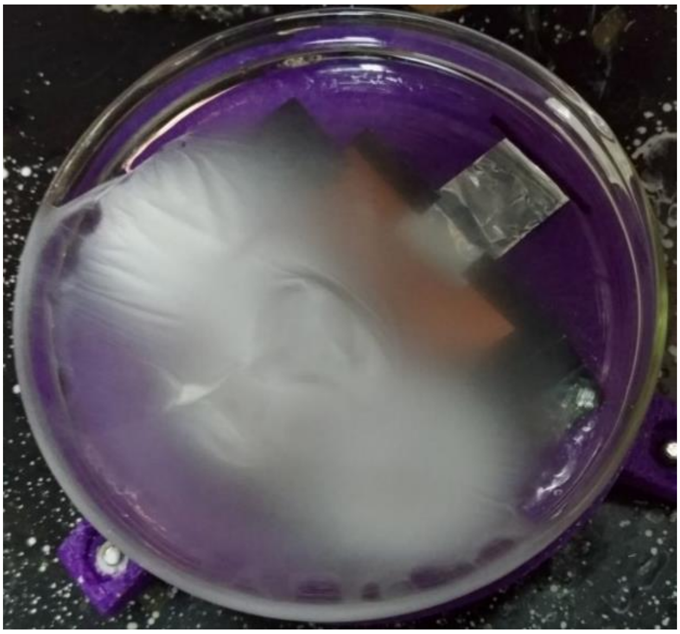

Figure S2. The fibers cover on the water surface when distance between the tip of the needle and the water surface was changed from 5 to 10 cm.

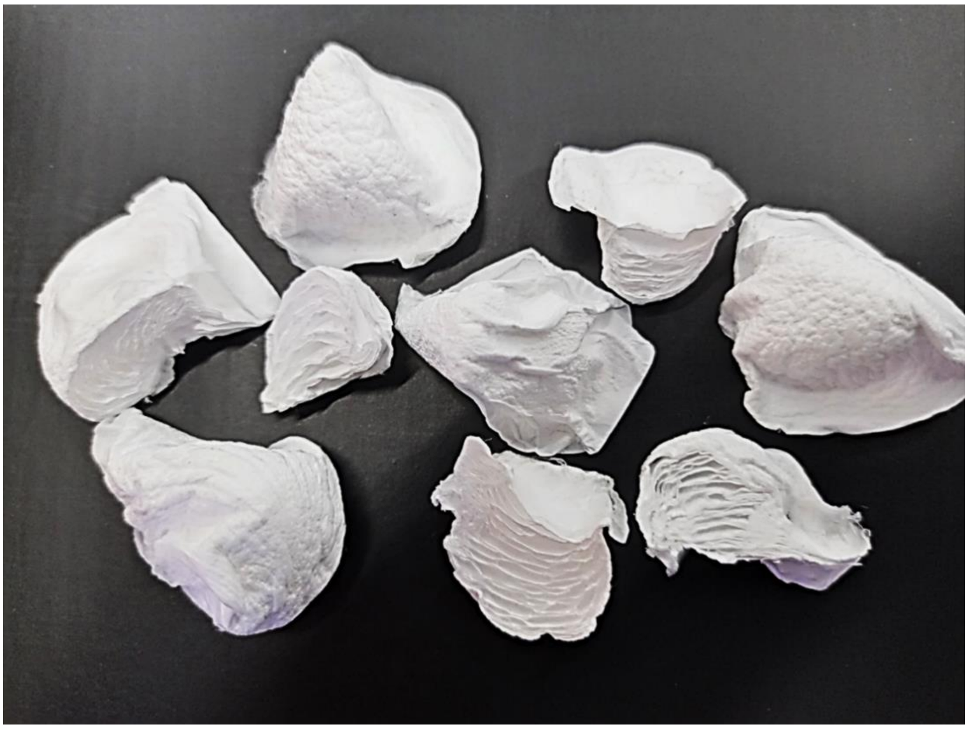

Figure S3. The termite nest-like 3D scaffold can be reproduced.

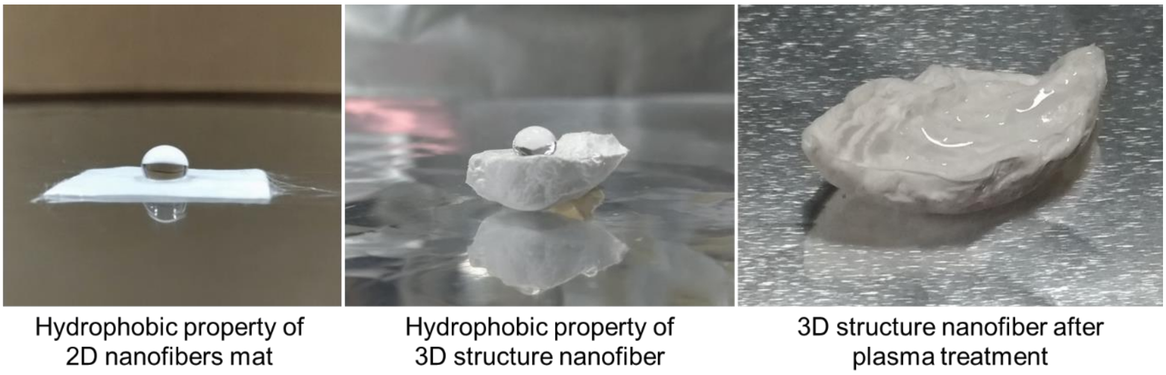

Figure S4. The water drops on the PVDF-HFP 2D and 3D nanofiber.

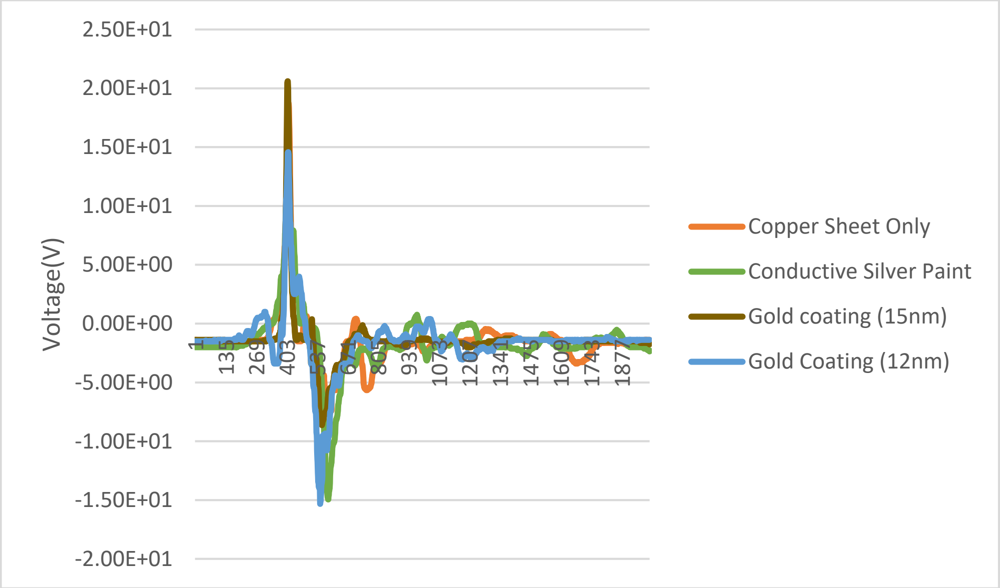

Figure S5. Open voltage response as a function of electrode attachment to the piezoelectric fibers.

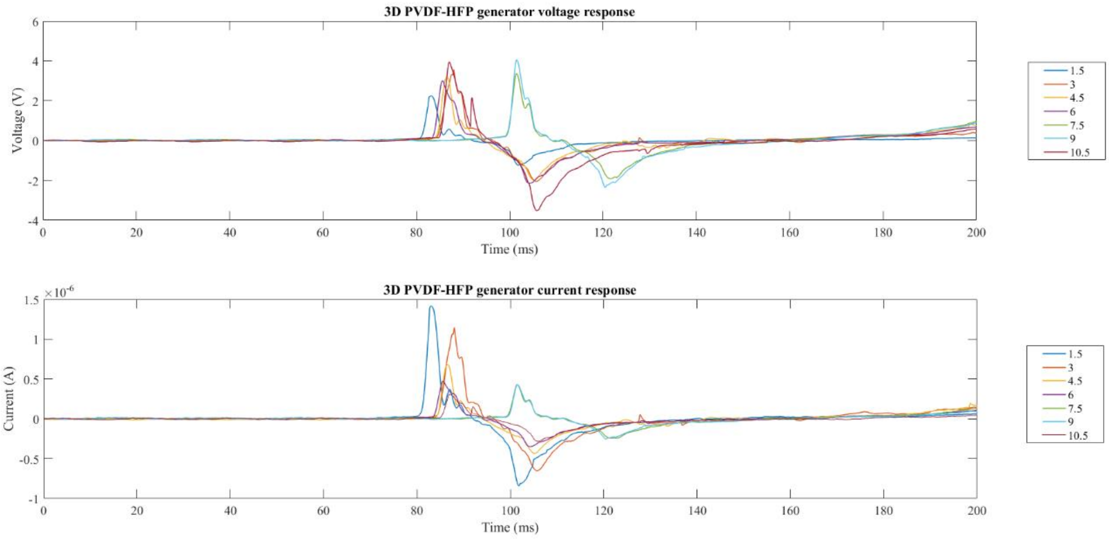

Figure S6. Voltage and current response of the 3D PVDF-HFP generator when a 100 g weight was dropped on its surface from a height of 5 cm and for different load conditions. The resistive loads used ranged from of 1.5 MΩ to 9 MΩ, in 1.5 MΩ increments.
